# Effect of Smoking Abstinence and Reduction in Asthmatic Smokers Switching to Electronic Cigarettes: Evidence for Harm Reversal

**DOI:** 10.3390/ijerph110504965

**Published:** 2014-05-08

**Authors:** Riccardo Polosa, Jaymin Morjaria, Pasquale Caponnetto, Massimo Caruso, Simona Strano, Eliana Battaglia, Cristina Russo

**Affiliations:** 1 Department of Clinical and Molecular Biomedicine, University of Catania, Catania 95125, Italy; E-Mails: p.caponnetto@unict.it (P.C.); mascaru@unict.it (M.C.); simostrano@hotmail.it (S.S.); eliana.battaglia@hotmail.it (E.B.); kristina_russo@yahoo.com (C.R.); 2 Centro per la Prevenzione e Cura del Tabagismo (CPCT), Teaching Hospital “Policlinico-V. Emanuele”, University of Catania, Catania 95123, Italy; 3 Department of Internal and Emergency Medicine, Teaching Hospital “Policlinico-V. Emanuele”, University of Catania, Catania 95123, Italy; 4 Department of Academic Respiratory Medicine, University of Hull, Castle Hill Hospital, Castle Road, Cottingham HU16 5JQ, UK; E-Mail: jbmorjaria@gmail.com

**Keywords:** smoking cessation, electronic cigarette, asthma, lung function, methacholine challenge, harm reduction

## Abstract

Electronic cigarettes (e-cigs) are marketed as safer alternatives to tobacco cigarettes and have shown to reduce their consumption. Here we report for the first time the effects of e-cigs on subjective and objective asthma parameters as well as tolerability in asthmatic smokers who quit or reduced their tobacco consumption by switching to these products. We retrospectively reviewed changes in spirometry data, airway hyper-responsiveness (AHR), asthma exacerbations and subjective asthma control in smoking asthmatics who switched to regular e-cig use. Measurements were taken prior to switching (baseline) and at two consecutive visits (Follow-up/1 at 6 (±1) and Follow-up/2 at 12 (±2) months). Eighteen smoking asthmatics (10 single users, eight dual users) were identified. Overall there were significant improvements in spirometry data, asthma control and AHR. These positive outcomes were noted in single and dual users. Reduction in exacerbation rates was reported, but was not significant. No severe adverse events were noted. This small retrospective study indicates that regular use of e-cigs to substitute smoking is associated with objective and subjective improvements in asthma outcomes. Considering that e-cig use is reportedly less harmful than conventional smoking and can lead to reduced cigarette consumption with subsequent improvements in asthma outcomes, this study shows that e-cigs can be a valid option for asthmatic patients who cannot quit smoking by other methods.

## 1. Introduction

Smoking prevalence in asthma is comparable to that found in the general population [1] and cigarette smoking is strongly predictive of the development of new-onset asthma in allergic adults [2]. It is increasingly recognized that the asthmatic smoker may represent a distinct disease phenotype with increased susceptibility of exacerbations and poor asthma-specific health status [3]. In fact it has been observed that a smoking history of more than 20 pack-years is associated with more severe disease [4]. Most studies report an accelerated decline in lung function and an increased severity of airflow obstruction in asthmatic smokers [5]. Moreover, asthma patients who smoke appear to have an impaired response to the beneficial effects of inhaled corticosteroids (ICS) or oral corticosteroids compared to asthmatics who do not smoke [6,7].

Of note, worsening asthma symptoms and lung function changes can be reversed with smoking cessation [8,9]. Unfortunately, there are only modest cessation rates reported in adult asthmatics [10] as there is a lack of awareness that smoking is a key aetiological component of their respiratory symptoms [11].

Electronic cigarettes (e-cigs) are battery-operated devices designed to vaporise nicotine without burning tobacco. These consumer products may be attractive to smokers who consider their tobacco use a recreational habit that they wish to maintain in a more benign form, rather than a predicament requiring medical treatment, and also to those smokers who cannot quit with current available medications due to their low efficacy. Indeed, e-cigs are increasingly being used as an alternative low risk substitute for conventional cigarettes [12,13].

Although these products have been shown to be effective and safe cigarette substitutes in randomized controlled trials of “healthy” smokers [14,15], no data is available regarding health effects of e-cigs use among vulnerable populations, including people with asthma. Moreover, it is unknown if regular “vaping” (the act of inhaling vapour from e-cigs/personal vaporizers) could result in improved asthma-related outcomes. Here we report, for the first time, changes in the level of asthma control, pulmonary function as well as airway hyperresponsiveness (AHR) in a group of asthmatic smokers who quit or reduced substantially their tobacco consumption by switching to e-cigs.

## 2. Methods

### 2.1. Patient Population

Medical records of patients with asthma regularly followed-up in our outpatient clinics were reviewed. From September 2012 to December 2013, a total of 18 asthmatic patients reporting regular use of e-cigs on at least two consecutive follow-up visits were identified. All patients had mild to moderate disease according to the Global Initiative for Asthma (GINA) criteria and treated accordingly with inhaled corticosteroids (ICS), long-acting (LABA) and on-demand short-acting β_2_ agonist (SABA) [16]. This study was approved by the local institutional ERB and informed consent was obtained from each patient.

### 2.2. Study Assessments

We obtained patient data from the clinic visit immediately preceding [baseline visit] the first of the two consecutive follow-up visits (follow-up visits 1 and 2). We also included data from the clinic visit immediately prior to the baseline visit (pre-baseline visit) to validate disease stability. In brief, data from four visits were collected and analysed. Pre-baseline visits were carried out at 6–12 months prior to baseline visits. Follow-up visits 1 and 2 were carried out at 6 (±1) and 12 (±2) months after baseline visits, respectively. At each clinic visit, patients were reassessed using a standard protocol consisting of clinical examination, review of smoking history, re-evaluation of treatment adherence and efficacy. The latter was assessed using: (i) Juniper’s Asthma Control Questionnaire (ACQ) score [17]; (ii) by annotating the number of exacerbations from the previous follow up visit (an asthma exacerbation was defined as an increase in respiratory symptoms requiring a short course of oral or parenteral corticosteroids); (iii) spirometry with parameters of forced expiratory flow in 1 second (FEV1), forced vital capacity (FVC), expiratory ratio (%FEV1/FVC) and forced expiratory flow at the middle half of the FVC (FEF25-75%); and (iv) in some subjects bronchial provocation tests assessing Airway HyperResponsiveness (AHR) with methacholine were also conducted as previously described [18].

### 2.3. Analyses

Baseline and demographic data were expressed as mean (±standard deviation (SD)) except for methacholine PC20 values expressed as geometric mean (data range). Post-initiation of e-cig data were similarly expressed. We also delineated data for single (e-cigs only) and dual users (e-cigs and conventional cigarettes). Statistical comparisons of parameters assessed were carried out using student’s T-test and Wilcoxon-signed rank test depending on whether the data was parametric or not, respectively. Missing measurements were not included in the analyses. A two-tailed *p* value of less than 0.05 was considered to indicate statistical significance. All analyses were performed with the Statistical Package for Social Science (SPSS for Windows version 18.0, IBM Inc., Chicago, IL, USA).

## 3. Results

### 3.1. Characteristics of the Patients and of Their E-cig Use

Of the 18 e-cig users identified (11 male, seven females) all were former tobacco smokers of about 20 conventional cigarettes/day. There were 10 single and eight dual users by the time of their most recent follow-up visit (follow-up visit 2). All dual users smoked ≤5 conventional cigarettes/day. The pre-baseline and baseline visit patient demographics and characteristics are summarised on Table 1. All patients initially switched to a cigarette-like model, but the majority went on to adopt a personal vaporizer. Duration of regular e-cig use ranged from 10 to 14 months, with twelve patients using them for more than a year. All patients took a stable dose of ICS, LABA as well as on-demand SABA throughout the observation period. None of the patients included had ever received a significant modification in anti-asthma therapy from their pre-baseline visit. There were no significant differences in the measured parameters of lung function, bronchial hyperresponsiveness (BHR) or ACQ scores between the pre-baseline and baseline visits (except for a small change in FEF25-75%) (Table 1).

**Table 1:** Patients characteristics at pre-baseline and baseline (before switching to e-cigs).

Parameter	Pre-baseline	Baseline		
	All Subjects (n = 18)	All Subjects (n = 18)	Single Users (n = 10)	Dual Users (n = 8)
Gender	11M, 7F	11M, 7F	7M, 3F	4M, 4F
Age	37.8 (±12.3)	38.8 (±12.3)	36.1 (±13.5)	42.3 (±10.6)
Asthma Duration	18.7 (±6.3)	19.7 (±6.3)	18.6 (±6.1)	21.0 (±6.8)
Conventional Cigarettes/Day	23.2 (±5.1)	21.9 (±4.5)	21.6 (±3.9)	22.4 (±5.3)
Smoking Pack Years	20.1 (±9.9)	21.0 (±10.7)	16.5 (±8.4)	26.6 (±11.2)
No. Exacerbations (from the previous follow up visit)	1.06 (±1.0)	1.17 (±0.9)	1.20 (±0.8)	1.13 (±1.0)
FEV1 (L)	3.35 (±0.76)	3.30 (±0.78)	3.42 (±0.84)	3.16 (±0.73)
FVC (L)	4.33 (±0.85)	4.28 (±0.90)	4.35 (±0.96)	4.19 (±0.88)
FEV1/FVC (%)	77.3 (±5.53)	76.8 (±4.52)	78.3 (±4.59)	75.0 (±3.98)
FEF25-75% (L/sec.)	2.86 (±0.69)	2.75 (±0.72)	2.95 (±0.53)	2.49 (±0.88)
ACQ	2.07 (±0.36)	2.03 (±0.37)	2.12 (±0.42)	1.93 (±0.29)
PC20 (mg/mL) *	1.31 (0.55, 1.75)	1.24 (0.49, 3.27)	1.10 (0.49, 2.07)	1.40 (0.82, 3.27)

Notes: n—number; M—male; F—female; mths—months; L–litres; L/sec—litres/second; %—percentage; mg/mL—milligram per milliliter; Data expressed as mean (±standard deviation); * Data expressed as geometric mean (range).

### 3.2. Efficacy of Smoking Reduction in Lung Function, BHR and ACQ Scores

**Figure 1:**
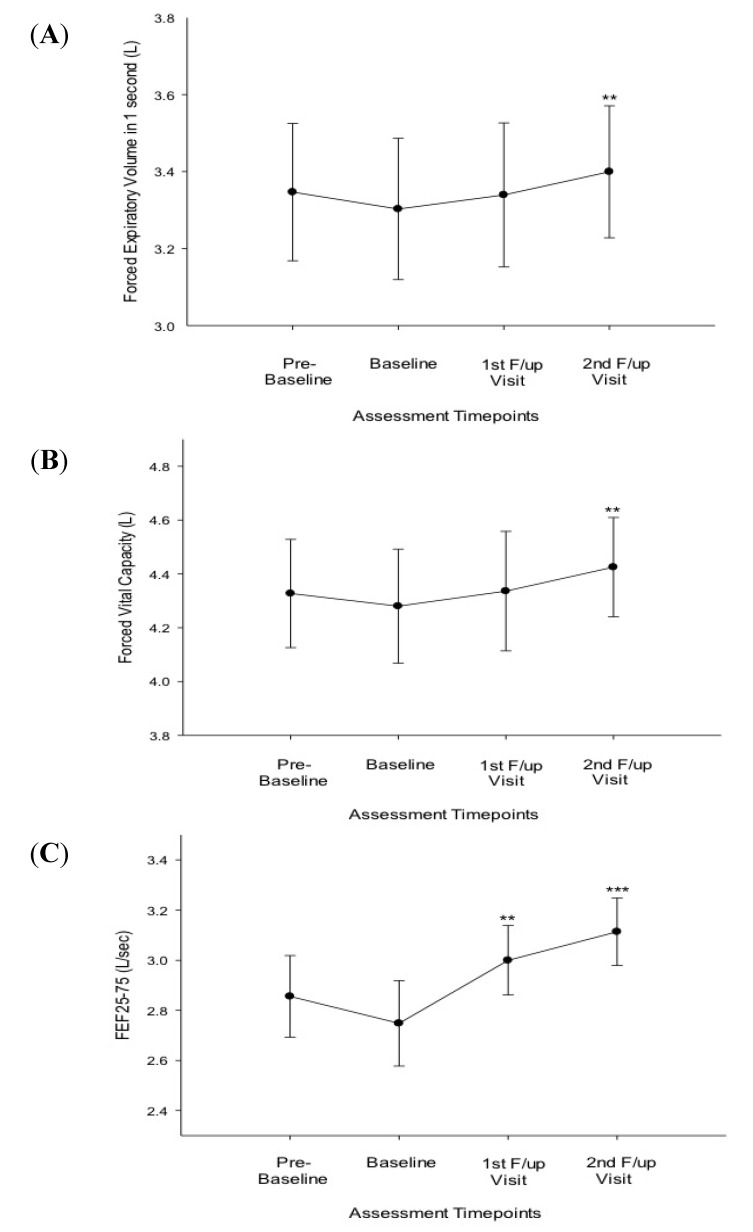
(**A**) Forced expiratory volume (FEV1) at the four timepoints of assessment for all 18 patients; (**B**) Forced vital capacity (FVC) at the four timepoints of assessment for all 18 patients; (**C**) Forced expiratory flow (FEF) 25–75 at the four timepoints of assessment for all 18 patients.

Notes: Compared to baseline significant *p* values of **—≤0.01, ***—≤0.001; All data expressed as mean and error bars are standard error of the mean; Abbreviations: F/up—follow-up; L—litres; L/sec.—litres per second.

**Figure 2:**
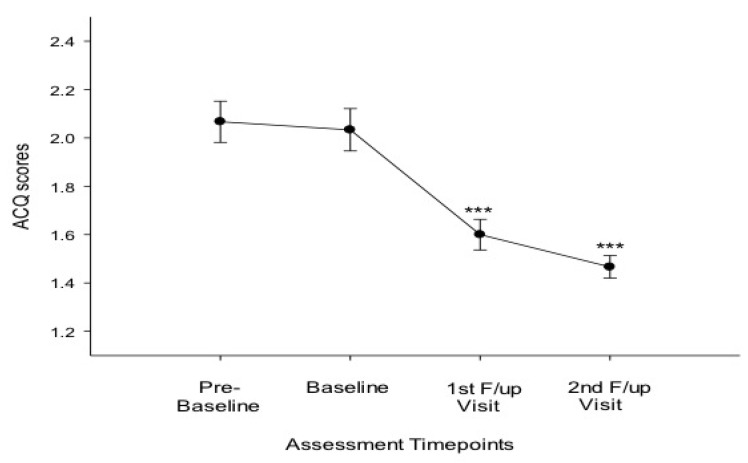
Asthma control questionnaire (ACQ) score at the four timepoints of assessment for all 18 patients.

Notes: Compared to baseline significant *p* values of ***—≤0.001; All data expressed as mean and error bars are standard error of the mean; Abbreviations: F/up—follow-up.

**Figure 3:**
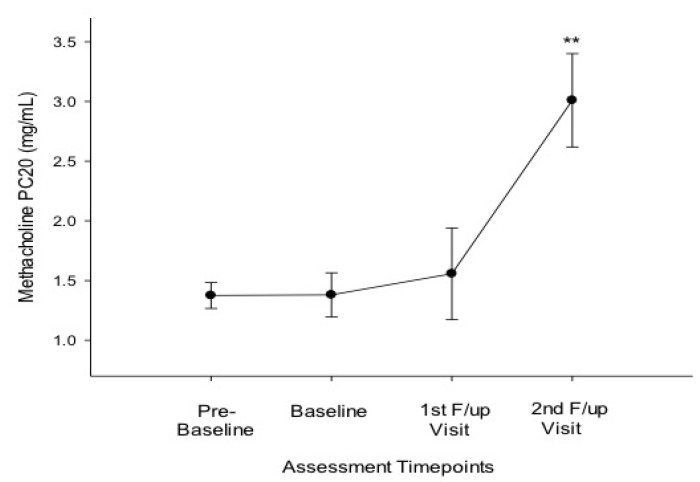
Methacholine PC20 at the four timepoints of assessment.

Notes: Compared to baseline significant *p* values of **—≤0.01. All data expressed as mean and error bars are standard error of the mean; Abbreviations: F/up—follow-up; mg/mL—milligram per millilitre.

Compared to baseline, at 6 months there were significant improvements in FEF25-75% (Table 2; Figure 1C) and ACQ scores (Table 2; Figure 2); at 12 months significant improvements were observed on all asthma outcomes measures, including methacholine PC20 (Table 2; Figure 1; Figure 2 and Figure 3). Dual users had similar changes to the overall group at 6 months (Table 2). At 12 months both dual and single users had considerable improvements compared to baseline in all parameters (except for FVC in single users) (Table 2).

### 3.3. Conventional Smoking

There was a marked reduction in conventional cigarette use amongst all e-cig users from a mean conventional cigarette/day use of 21.9 at baseline decreasing to 1.7 at follow-up visit 2 (*p* < 0.001) (Table 2). Similar substantial reduction in conventional cigarette smoking was observed in dual users as well (22.4 at baseline to 3.9 at follow-up visit 2; *p* < 0.001) (Table 2). Importantly, 10 asthmatics gave up conventional cigarette use in favour of the e-cig (single users).

### 3.4. Exacerbations

Prior to e-cig use in the 18 patients the average number of exacerbations were 1.06 (at pre-baseline) and 1.17 (at baseline) (Table 2). Over the period of observation none of the subjects in the cohort reviewed had a hospital or intensive care unit admission. At follow-up visits 1 (0.87 exacerbations) and 2 (0.78 exacerbations) although there was a reduction in exacerbations compared to baseline which was not significant (*p* = 0.296 and 0.153, respectively) the reduction in exacerbations was marked (>25 and >33 %, respectively) (Table 2).

**Table 2:** Changes in parameters measured at baseline, 1st- and 2nd follow-up visits.

Parameter	Baseline	1st Follow-up Visit (6 months ± 1)		2nd Follow-up Visit (12 months ± 2)	
		*p* value to Baseline		*p* value to Baseline	
**All patients (n = 18; 11M, 7F)**					
FEV1 (L)	3.30 (±0.78)	3.34 (±0.72)	0.078	3.40 (±0.73)	0.005
FVC (L)	4.28 (±0.90)	4.34 (±0.86)	0.105	4.43 (±0.78)	0.006
FEF25-75% (L/sec.)	2.75 (±0.72)	3.00 (±0.54)	0.006	3.11 (±0.57)	0.001
ACQ	2.03 (±0.37)	1.60 (±0.24)	<0.001	1.47 (±0.20)	<0.001
PC20 (mg/mL) *	1.24 (0.49, 3.27)	1.20 (0.44, 4.23)	0.594	2.56 (0.5, 5.55)	0.003
Cigarettes/day	21.9 (±4.5)	1.9 (±2.9)	<0.001	1.7 (±2.1)	<0.001
Exacerbations	1.17 (±0.9)	0.87 (±0.7)	0.296	0.78 (±0.7)	0.153
**Single Users (n = 10; 7M, 3F)**					
FEV1 (L)	3.42 (±0.84)	3.49 (±0.75)	0.779	3.51 (±0.75)	0.032
FVC (L)	4.35 (±0.96)	4.52 (±0.86)	0.161	4.51 (0.81)	0.059
FEF25-75% (L/sec.)	2.95 (±0.53)	3.17 (±0.39)	0.093	3.20 (±0.54)	0.047
ACQ	2.12 (±0.42)	1.69 (±0.29)	0.024	1.50 (±0.19)	<0.001
PC20 (mg/mL) *	1.10 (0.49, 2.07)	1.14 (0.44, 3.55)	0.463	2.06 (0.5, 5.55)	0.043
Cigarettes/day	21.6 (±3.9)	-	-	-	-
Exacerbations	1.20 (±0.8)	1.13 (±0.6)	0.831	1.10 (±0.7)	0.773
**Dual Users (n = 8; 4M, 4F)**					
FEV1 (L)	3.16 (±0.73)	3.17 (±0.71)	0.062	3.26 (±0.72)	0.05
FVC (L)	4.19 (±0.88)	4.13 (±0.88)	0.352	4.32 (±0.78)	0.05
FEF25-75% (L/sec.)	2.49 (±0.88)	2.81 (±0.64)	0.028	3.01 (±0.62)	0.012
ACQ	1.93 (±0.29)	1.50 (±0.15)	0.004	1.43 (±0.21)	0.002
PC20 (mg/mL) *	1.40 (0.82, 3.27)	1.28 (0.72, 4.23)	0.893	3.17 (2.39, 4.09)	0.028
Cigarettes/day	22.4 (±5.3)	5.0 (±2.6)	<0.001	3.9 (±1.0)	<0.001
Exacerbations	1.13 (±1.0)	0.57 (±0.8)	0.257	0.38 (±0.5)	0.078

Notes: n—number; M—male; F—female; L –litres; %—percentage; L/sec.—litres per second; mg/mL—milligram per milliliter; Data expressed as mean (±standard deviation); *Data expressed as geometric mean (range).

### 3.5. Safety and Tolerability

No severe adverse reactions or acute exacerbation of asthma symptoms (*i.e.*, cough, wheeze) were reported during period of observation with e-cig use. Overall, e-cig use appears to be well tolerated in these asthmatic patients with dry mouth and throat irritation being occasionally reported.

## 4. Discussion

Here, we show for the first time improvements in asthma control, AHR and pulmonary function in asthmatic smokers who quit or dramatically reduced their tobacco consumption by switching to e-cigs.

These preliminary findings are of great consequence considering that many asthmatic patients continue to smoke and seem uninterested in quitting [3,8], a paradox that may be explained by the highly addictive nature of tobacco smoking and the remitting clinical nature of asthma, particularly in its mild-to-moderate forms. The success with e-cig observed in these patients may be explained by the great compensatory effect of e-cigs at both physical and behavioural level [12]; in particular these products are known to provide a coping mechanism for conditioned smoking cues by replacing some of the rituals associated with smoking gestures (e.g., hand-to-mouth action of smoking). In agreement with this, we have demonstrated that nicotine free plastic inhalators can only improve quit rates in those smokers for whom it was shown that cigarette handling and manipulation played an important role in their smoking ritual [18].

This study has demonstrated that lung function of smokers with asthma may improve when stopping smoking for a sufficient period of time. The level of improvement was small and likely to be clinically irrelevant, but statistically significant. In asthma, there have been two previous studies looking at the effect of stopping smoking on lung function [8,9], and our retrospective findings are in agreement with the positive results of these prospective studies. Taken together, the evidence suggests that the harmful effects of smoking on the asthmatic airways can be reversed. The improvement in lung function could be due to a reduction in the pro-inflammatory effects of cigarette smoke on the airways [3,19]. It is also possible that stopping smoking leads to a reduction in corticosteroid insensitivity, as smoking asthmatic patients appear to be less sensitive to the beneficial effects of inhaled corticosteroids (ICSs) on lung function compared with non-smoking asthmatic patients [6,20,21] and stopping smoking by switching to e-cigs may allay this unresponsiveness to ICSs.

It was interesting to note an early improvement in FEF25–75% values in the asthmatic smokers who successfully quit smoking with e-cigs use, whereas no significant change was detected in FEV1 or FVC at the first follow-up visits immediately after e-cig switch. Larger clinical trials are required to shed more light on the significance of these findings in relation to previous smoking history and to current vaping behaviour.

There were significant improvements in BHR, in the asthmatic smokers who had been abstinent or reduced their tobacco consumption long enough. The level and time-course of the observed changes in PC20 methacholine are not unexpected and consistent with the results of prospective studies in allergic subjects for whom an objective proof of cessation was documented [22]. The observed improvement in BHR may have important clinical implications. For instance, it is well known that, independent of smoking status, individuals with documented AHR are known to be at risk for asthma symptoms and attenuated pulmonary function levels [23,24]. Thus, improving AHR may confer some clinical benefit as documented in the asthmatic patients of this study. The explanation for the improvement in AHR is unknown, but probably relates to the gradual and progressive reduction in smoke-induced inflammatory changes on the airways of those who quit and/or to the reversal of corticosteroid insensitivity [3,19].

We have also observed early and stable improvements in asthma control (*i.e*., ACQ scores) that are clinically relevant. This is not surprising considering the established relationship between smoking and poor asthma control reported in population-based surveys [25,26] and controlled studies [4,27].

In spite of significant improvement in lung function, AHR and asthma control, no significant change in disease exacerbations was observed. This discrepancy can be explained by the low baseline values for exacerbations in our mild-to-moderate asthmatic patient cohort (hence it is possible that scope for further improvement was limited), the small sample size, and the modality of documenting exacerbations which was liable to recall bias.

Of interest, consistent improvements in subjective and objective asthma outcomes were also observed amongst dual users (*i.e.*, heavy reducers) with no real difference in dual compared to single users by the end of the observation period. Surprisingly, early improvement in FEF25–75% values were shown only in dual users (unlike in single users). This could be due to substantial differences in the level of exposure to tobacco smoke between single and dual users (mean pack/years 16.5 in single *vs.* 26.6 in dual users) and, consequently, to the much higher FEF25–75% values at baseline in single compared to dual users (thus limiting room for further improvement in single users). An alternative explanation is that the observed early improvement in FEF25–75% values could be the result of a chance finding given the small sample size.

It is impossible to know for sure whether e-cig use drove the observed positive changes in asthma outcomes, but clearly was not reported to be harmful in mild-to-moderate asthmatics in this study. None of the patients reported severe adverse reactions or acute exacerbation of asthma symptoms during e-cig use. Their use appears to be well tolerated with occasional dry mouth and throat irritation being reported. The current evidence indicates that e-cigs are by far a less harmful alternative to tobacco smoking and have a potential significant health benefit in smokers who switch to these products [28].

By substantially reducing number of cigarettes smoked per day and exposure to their hazardous toxicants, e-cigs may not only improve asthma symptoms and pulmonary function but may also confer an overall health advantage in smokers with asthma [13]. Therefore, e-cig use in asthmatic smokers unable or unwilling to quit should be exploited as a safer alternative approach to harm-reversal (*i.e.*, specific reversal of asthma-related outcomes) and, in general, to harm-reduction (*i.e.*, overall reduction of smoke-related diseases).

There are some limitations in our study. Firstly, this is a small retrospective study, hence the results observed may be due to bias and not due to a true effect; and consequently be interpreted with caution. However, despite being a small study we were able to detect positive significant changes for several asthma outcomes. Standard issues associated with retrospective studies (including variance in the quality of information recorded by medical professionals and difficulty in establishing a causal relationship) need consideration. Nonetheless, a clear advantage of conducting such studies is that they permit the generation of hypotheses that then would be tested prospectively under controlled conditions. Secondly, patients in this study may represent a self-selected sample, which is not representative of all asthmatic smokers who switch to e-cigs. Thirdly, assessment of symptoms in our patients was not rigorous and liable to recall bias. Although it is unlikely that the experience of troublesome symptoms upon switching may have been easily forgotten, the good tolerability reported by these patients should be also considered with prudence. Lastly, smoking abstinence was self-reported. However, self-reported number of cigarettes smoked per day in studies of this type is not subjected to the kind of biases observed in clinical trials where there is the tendency to claim abstinence [29]. Moreover, similar beneficial effects were also reported in dual users (*i.e*., smoking reducers) and therefore objective measures of abstinence are unlikely to be of great importance.

## 5. Conclusions

The e-cig may help smokers with asthma to reduce their cigarette consumption or remain abstinent and hence reduce the burden of smoking-related asthma symptoms. The positive findings observed with e-cigs allows us to advance the hypothesis that these products may be valuable for smoking cessation and/or tobacco harm reduction also in asthma patients who smoke. Large randomized controlled trials are now needed to confirm and expand these preliminary observations.
